# No disease left behind

**DOI:** 10.18632/oncotarget.28700

**Published:** 2025-03-13

**Authors:** Muzamil Arshad, Connor Lynch, Rohan R. Katipally, Sean P. Pitroda, Ralph R. Weichselbaum

**Keywords:** SBRT, ablation, immunotherapy, Radiothreapy, Oncology

## INTRODUCTION

High-dose radiotherapy is considered curative due to high radiographic local control rates. Herein, we review the limitations of response assessment and explore the oncological consequences of residual disease after radiotherapy.

### Is SABR ablative?

**Figure 1:**
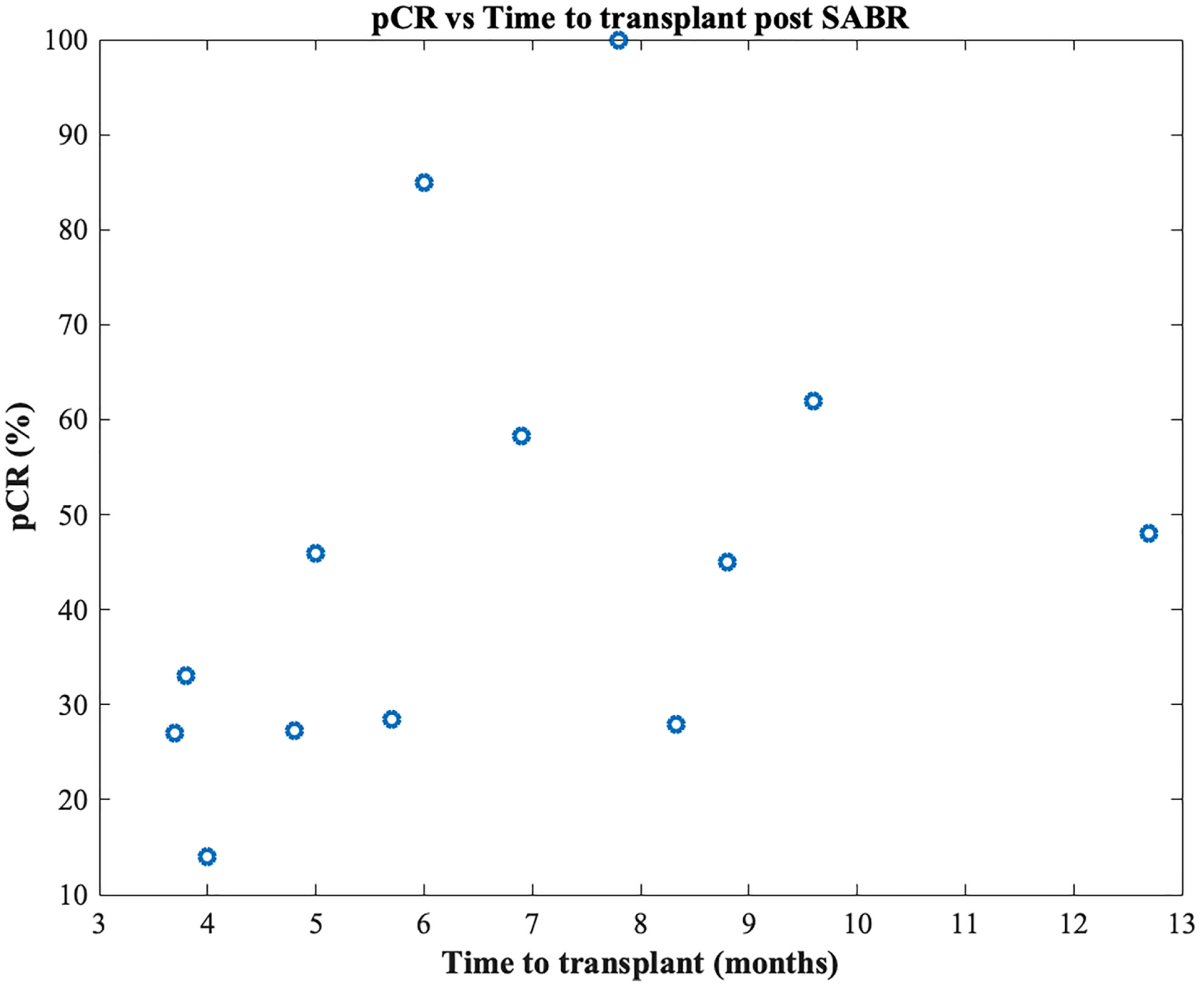
Pathological complete response rates versus time to transplant after SABR. Each data point represents a different study. There is no association between time to transplant after SABR and pCR rate (R^2^ = 0.16, *p* = 0.18).

Stereotactic ablative radiotherapy (SABR) is utilized in various settings including non-operative early-stage non-small cell lung cancer (NSCLC), prostate adenocarcinoma, hepatocellular carcinoma (HCC), renal cell carcinoma (RCC) and in the metastatic setting. SABR is considered ablative due to excellent radiographic local control rates. Three-year radiographic control rates in early-stage NSCLC [1], renal cell carcinoma [2] and hepatocellular carcinoma [3] are 85+%, 100% and 86% respectively, and 5-year biochemical control rates of 85+% are seen in prostate cancer [4]. Pathological analysis, however, shows SABR is potentially *not* ablative. Residual cancer is identified on histology in 40% of lung [5], 57–69% of renal cell [6, 7], 7.7–47.6% of prostate [8] and 0–86.7% [9–22] of hepatocellular carcinoma. Also, there is no increase in pathological complete response (pCR) rates with increasing time following SABR (<74 vs. >74 days) in lung cancer [5, 23] or in hepatocellular carcinoma (Table 1 and Figure 1). Due to this broad range of response rates, controversy exists over the meaning of residual disease on pathological examination following treatment [24].

**Table 1:** Pathological outcomes after SABR

Tumor diameter (cm)	BED10	Time to transplant (months)	pCR (%)
4.5 [9]	58.83	Not Reported	13.3
2.01 [10]	47.6	4	14
2.67 [11]	168.7^b^	3.8^a^	25
3 [12]	137.7	3.7^a^	27
2.5 [13]	151.2	4.8	27.3
3.2 [14]	85.5	8.33^b^	28
3 [15]	100	5.7	28.5
2.4 [16]	72	8.8	45
2.3 [17]	72	5	46
2.8 [18]	100	12.7^b^	48.1
2.6 [19]	85.5	6.9	58.3
3.05 [20]	100	9.6^a^	62
2.8 [21]	168	6	85
4.2 [22]	112.5	7.8	100^c^

^a^Time was reported in days in paper, converted to months in this table. ^b^Calculated from reported data in paper. ^c^1–2 cycles of TACE was given prior to SABR.

Discordance between pCR and radiographic local control rates is multi-factorial. While radiographic rates of local control are high, they may be artificially elevated because of limited follow-up, high rates of mortality, and small patient sample sizes. Local failures can often be difficult to evaluate in the background of post-SABR changes such as lung fibrosis or consolidation. The 3-year radiographic local control rates for liver metastases ranges between 50–91% [25, 26] and for pancreatic cancer, 58.5% [27]. Inferior outcomes in these settings may be related to the inability of SABR to achieve ablation. Here, we review the potential oncological significance of residual disease and discuss potential strategies to improve the ablative potential of SABR.

### Residual disease is associated with worse outcomes

Treatment response is assessed by various approaches, including physical exam (e.g., endoscopy), radiology (anatomic or metabolic imaging), blood-based biomarker (prostate specific antigen), or invasively with histological assessment of residual disease. A clinical complete response (cCR) is generally defined as no visible disease on radiographic or physical examination, whereas pathological complete response (pCR) is defined as no histological evidence of tumor. Anal squamous cell carcinoma (SCC) treated with chemoradiation demonstrates delayed tumor regression with cCR at 11 weeks in 53–73% of patients which increases to 80–90% at 26 weeks [28]. Pathological complete response from biopsies between 4–6 weeks is 83–93% depending on tumor size [29]. While delay in clinical response/tumor regression is *not* associated with inferior oncological outcomes, patients without a cCR at 6 months have worse overall survival compared to those with cCR (5-year survival 46 vs. 87%, respectively) [28].

Rectal adenocarcinomas treated with total neoadjuvant therapy (TNT) with long-course chemoradiotherapy exhibit a cCR of 41% at a median of 7.6 weeks without significant clinical regression thereafter [30]. pCR is 28% with TNT [31]. Unlike anal cancer, patients who don’t achieve a cCR at initial assessment have worse outcomes. Clinical local failure rate is 50% vs. 20% respectively if <cCR vs. cCR. More concerning is that distant metastasis (DM), among those with an initial cCR who ultimately develop local failure (LF), is 22.2% vs. 5.2% for those with sustained response [30]. The Memorial Sloan Kettering Cancer Center (MSKCC) watch-and-wait experience in non-operative management of rectal cancer confirms the negative prognostic value of residual disease. Among patients with pCR after neoadjuvant therapy and surgery, 5-year OS/DFS is 94/92% versus 73/75% for those with a cCR [32].

Residual disease is similarly prognostic in other malignancies. Patients treated with neoadjuvant chemoradiotherapy for cervical cancer demonstrate inferior outcomes based on the extent of residual disease. Disease-free and overall survival at 5 years for patients with pCR, microscopic residual disease (<3 mm) and macroscopic residual disease (>3 mm) was approximately 86/92%; 80/89%; 56/68% [33]. Using individual patient data from randomized trials and Markov chain modeling, Kishan et al. demonstrate that failure to control local disease may seed distant metastasis in prostate cancer [34]. This is consistent with the MSKCC experience showing increased risk of distant metastasis in patients with residual disease on prostate biopsy (at median of 38 months post radiotherapy), 45% vs. 19% at 15 years [35]. Similar results were seen in patients treated with SABR for prostate adenocarcinoma with 5-year PSA relapse of 57% vs. 7% for those with residual disease versus those with pCR respectively (biopsy performed at median of 2.2 years post SABR) [36].

Collectively, these data demonstrate that across a variety of cancers residual disease can persist after therapy and is associated with worse outcomes. These findings are consistent across treatment strategies (conventionally fractionated radiotherapy, chemoradiotherapy and SABR).

### Dose escalation and novel combination therapies

While worse disease biology may increase the likelihood of both local failure and distant metastasis, accounting for the association between residual disease and worse outcomes, multiple studies have shown that dose escalation improves outcomes. The OPERA trial showed improved rectal preservation rates for tumors <3 cm with dose escalation using contact brachytherapy (63% vs. 97% at 3 years) [37]. A phase I SABR dose-escalation study for localized prostate cancer showed 2-year positive biopsies decreased with dose-escalation: 47.6%, 19.2%, 16.7% and 7.7% for 32.5, 35, 37.5 and 40 Gy with corresponding 5/8-year PSA failure: 15/26%, 6/15%, 0/3.4% and 0/6% respectively [8, 38]. The phase III FLAME trial confirmed that dose-escalated treatment of intra-prostatic nodules improves biochemical control (85% vs. 92% at 5 years) [39].

One phase III trial in intermediate risk prostate cancer (RTOG 0126) demonstrated reduced distant metastasis at 15 years (11 vs. 6%) [40] with prostate radiation dose escalation using conventional fractionation. Similarly, in high-risk patients GETUG 18 showed improved overall survival, cancer-specific survival, and progression-free survival with dose escalation to the prostate [41]. Retrospective data has demonstrated dose escalated SABR (BED_10_ >100 Gy) improves radiographic local control rates for colorectal liver metastasis (93% vs. 65% at 3 years) [26].

Androgen deprivation therapy and chemotherapy are well-established systemic therapies known to improve response to radiotherapy and oncological outcomes. Immune checkpoint inhibitors have shown efficacy for a variety of cancers, particularly in the low metastatic burden setting [42], and combining this with radiotherapy is gaining interest. The phase II randomized trial by Chang et al. compared SABR alone vs. SABR + nivolumab (1st cycle delivered same day or 36 hours after first fraction, q4 weeks for each cycle) and found that combination therapy decreased radiographic local failures (13.3% vs. 0%) and distant metastasis (16% vs. 3%) [43]. Radiation dose escalation and use of checkpoint inhibitors have not uniformly produced improved outcomes however (ARTDECO, RTOG 0617, CALLA) [44–46]. Future trials should explore a combination of dose escalation, immune modulators to decrease the immune suppressive effects of radiotherapy and checkpoint inhibitors to improve the ablative potential of SABR.

## CONCLUSIONS

SABR is a well-established therapy in both the curative and metastatic setting, however, its ablative potential may not be as high as suggested by radiographic local control rates. Post-SABR biopsies reveal nontrivial rates of residual disease, which is associated with worse outcomes. Radiation dose-escalation and novel immune modulating systemic therapies may improve the ablative potential of SABR and ultimately translate to improved oncological outcomes.

## References

[1] Tsang MW . J Thorac Dis. 2016; 8:S517–27. 10.21037/jtd.2016.03.14. 27606082 PMC4990666

[2] Siva S , et al. Lancet Oncol. 2022; 23:1508–16. 10.1016/S1470-2045(22)00656-8. 36400098

[3] Gerum S , et al. J Gastrointest Oncol. 2024; 15:1880–92. 10.21037/jgo-23-771. 39279965 PMC11399857

[4] van As N , et al. N Engl J Med. 2024; 391:1413–25. 10.1056/NEJMoa2403365. 39413377 PMC7616714

[5] Palma DA , et al. JAMA Oncol. 2019; 5:681–88. 10.1001/jamaoncol.2018.6993. 30789648 PMC6512269

[6] Tang C , et al. Lancet Oncol. 2021; 22:1732–39. 10.1016/S1470-2045(21)00528-3. 34717797 PMC11975425

[7] Swaminath A , et al. Int J Radiat Oncol Biol Phys. 2023; 117:S82. 10.1016/j.ijrobp.2023.06.402.

[8] Zelefsky MJ , et al. Int J Radiat Oncol Biol Phys. 2019; 104:42–49. 10.1016/j.ijrobp.2018.12.045. 30611838 PMC7525798

[9] Sapisochin G , et al. J Hepatol. 2017; 67:92–99. 10.1016/j.jhep.2017.02.022. 28257902

[10] Facciuto ME , et al. J Surg Oncol. 2012; 105:692–98. 10.1002/jso.22104. 21960321

[11] O’Connor JK , et al. Liver Transpl. 2012; 18:949–54. 10.1002/lt.23439. 22467602

[12] Moore A , et al. Radiat Oncol. 2017; 12:163. 10.1186/s13014-017-0899-4. 29052532 PMC5649060

[13] Uemura T , et al. World J Surg. 2019; 43:886–93. 10.1007/s00268-018-4829-x. 30361748

[14] Mohamed M , et al. Adv Radiat Oncol. 2015; 1:35–42. 10.1016/j.adro.2015.12.003. 28799575 PMC5506745

[15] Bauer U , et al. World J Gastroenterol. 2021; 27:3630–42. 10.3748/wjg.v27.i24.3630. 34239274 PMC8240047

[16] Mannina EM , et al. Int J Radiat Oncol Biol Phys. 2017; 97:931–38. 10.1016/j.ijrobp.2016.12.036. 28333015

[17] Gresswell S , et al. J Radiosurg SBRT. 2018; 5:261–67. 30538886 PMC6255717

[18] Wong TC , et al. Hepatology. 2021; 74:2580–94. 10.1002/hep.31992. 34091914 PMC9291538

[19] Lee VH , et al. JAMA Netw Open. 2024; 7:e2415998. 10.1001/jamanetworkopen.2024.15998. 38857045 PMC11165380

[20] Garg R , et al. Adv Radiat Oncol. 2020; 6:100559. 10.1016/j.adro.2020.08.016. 33665482 PMC7897771

[21] Gerum S , et al. Strahlenther Onkol. 2020; 196:334–48. English. 10.1007/s00066-019-01540-8. 31732784

[22] Jacob R , et al. Liver Transpl. 2016; 22:547–51. 10.1002/lt.24398. 26785388 PMC4809754

[23] Roy SF , et al. Transl Lung Cancer Res. 2019; 8:S124–34. 10.21037/tlcr.2019.09.05. 31673516 PMC6795577

[24] Correa RJM , et al. Eur Urol. 2023; 84:287–88. 10.1016/j.eururo.2023.03.025. 37032187

[25] Méndez Romero A , et al. Int J Radiat Oncol Biol Phys. 2021; 109:1377–86. 10.1016/j.ijrobp.2020.11.045. 33451857

[26] Ohri N , et al. Int J Radiat Oncol Biol Phys. 2021; 110:188–95. 10.1016/j.ijrobp.2017.12.288. 29395629 PMC6102100

[27] Comito T , et al. Curr Oncol. 2023; 30:7073–88. 10.3390/curroncol30070513. 37504373 PMC10378012

[28] Glynne-Jones R , et al. Lancet Oncol. 2017; 18:347–56. 10.1016/S1470-2045(17)30071-2. 28209296 PMC5337624

[29] Flam M , et al. J Clin Oncol. 1996; 14:2527–39. 10.1200/JCO.1996.14.9.2527. 8823332

[30] Thompson HM , et al. JAMA Netw Open. 2024; 7:e2350903. 10.1001/jamanetworkopen.2023.50903. 38194231 PMC10777257

[31] Conroy T , et al. Lancet Oncol. 2021; 22:702–15. 10.1016/S1470-2045(21)00079-6. 33862000

[32] Smith JJ , et al. JAMA Oncol. 2019; 5:e185896. 10.1001/jamaoncol.2018.5896. 30629084 PMC6459120

[33] Federico A , et al. Ann Surg Oncol. 2022; 29:4806–14. 10.1245/s10434-022-11583-4. 35355131 PMC9246767

[34] Kishan AU , et al. Eur Urol. 2020; 77:201–8. 10.1016/j.eururo.2019.10.008. 31718822 PMC7008470

[35] Zelefsky MJ , et al. J Urol. 2019; 201:1127–33. 10.1097/JU.0000000000000110. 30741847 PMC7543653

[36] Zelefsky MJ , et al. Radiother Oncol. 2021; 159:33–38. 10.1016/j.radonc.2021.02.008. 33587971 PMC10187562

[37] Baron D , et al. Ann Oncol. 2025; 36:208–15. 10.1016/j.annonc.2024.10.827. 39532203

[38] Moore A , et al. Eur Urol Oncol. 2024; 7:812–20. 10.1016/j.euo.2023.10.019. 37949730 PMC13435800

[39] Kerkmeijer LGW , et al. J Clin Oncol. 2021; 39:787–96. 10.1200/JCO.20.02873. 33471548

[40] Michalski JM , et al. Int J Radiat Oncol Bio Phys. 2023; 117:S4–5. 10.1016/j.ijrobp.2023.06.210.

[41] Hennequin C , et al. J Clin Oncol. 2024; 42. 10.1200/JCO.2024.42.4_suppl.LBA259.

[42] Lynch C , et al. J Clin Oncol. 2024; 42:3387–91. 10.1200/JCO.24.00549. 39038267 PMC11458364

[43] Chang JY , et al. Lancet. 2023; 402:871–81. 10.1016/s0140-6736(23)01384-3. 37478883 PMC10529504

[44] Hulshof MCC , et al. J Clin Oncol. 2021; 39:2816–24. 10.1200/JCO.20.03697. 34101496

[45] Bradley JD , et al. J Clin Oncol. 2020; 38:706–14. 10.1200/JCO.19.01162. 31841363 PMC7048161

[46] Monk BJ , et al. Lancet Oncol. 2023; 24:1334–48. 10.1016/S1470-2045(23)00479-5. 38039991

